# Utility of magnetic resonance imaging in determining treatment response and local recurrence in nasopharyngeal carcinoma treated curatively

**DOI:** 10.1186/s12885-020-6664-3

**Published:** 2020-03-06

**Authors:** Katherine Meng, Jeremy Tey, Francis Cho Hao Ho, Hira Asim, Timothy Cheo

**Affiliations:** Department of Radiation Oncology, National University Cancer Institute, 5 Lower Kent Ridge Road, Singapore, 119074 Singapore

**Keywords:** Nasopharyngeal carcinoma, Nasopharyngeal neoplasms, Radiotherapy, Intensity-modulated radiotherapy, Magnetic resonance imaging

## Abstract

**Background:**

To determine the optimal timing of the first Magnetic Resonance Imaging (MRI) scan after curative-intent radiotherapy (RT) for nasopharyngeal carcinoma (NPC), and evaluate the role of MRI in surveillance for locoregional recurrence (LRR).

**Methods:**

Patients with non-metastatic NPC treated radically who had at least one post-treatment MRI (ptMRI) done were included for analysis. ptMRI reports were retrospectively reviewed and categorised as complete response (CR), partial response/residual disease (PR) or indeterminate (ID). Patients with LRR were assessed to determine if initial detection was by MRI or clinical means. Univariable and multivariable Cox proportional hazard regression analysis were performed to identify independent factors associated with CR on ptMRIs.

**Results:**

Between 2013 and 2017, 262 eligible patients were analysed, all treated with Intensity Modulated Radiotherapy (IMRT). Median time from end of RT to the first ptMRI was 93 days (range 32–346). Of the first ptMRIs, 88 (33.2%) were CR, 133 (50.2%) ID, and 44 (16.6%) PR. A second ptMRI was done for 104 (78.2%) of 133 patients with ID status. In this group, 77 (57.9%) of the subsequent MRI were determined to be CR, 21(15.8%) remained ID and 6 (4.5%) PR. T1 tumour stage and AJCC stage I were associated with increased CR rates on first ptMRI on multivariable analysis. ID status was more likely at 75–105 days (3 months +/− 15 days) vs 106–135 days (4 months +/− 15 days) post RT (OR 2.13, 95% CI 1.16–4.12, *p* = 0.024). LRR developed in 27 (10.1%) patients; 20 (74.1%) were initially detected through MRI, 3 (11.1%) by nasoendoscopy and 2 (7.4%) by PET-CT.

**Conclusion:**

MRI is useful for detecting local recurrence or persistent disease after curative-intent treatment. Most patients will need more than one ptMRI to arrive at a definitive status. The rate of ID ptMRI may be reduced by delaying the first scan to around 4 months post RT.

## Background

Nasopharyngeal Carcinoma (NPC) has a distinct ethnic and geographical distribution, and is common in Southern Chinese and South East Asian populations [1]. Non-metastatic NPC is treated definitively with radiotherapy (RT) with or without chemotherapy. Treatment response has been closely associated with prognosis [2, 3]. Magnetic Resonance Imaging (MRI)‘s role in the initial staging of biopsy-proven NPC is well established [4–7]. However, the utility of MRI in the assessment of response to treatment and disease surveillance is less well-defined.

The time course of histological NPC regression has previously been demonstrated through serial biopsy to be around 12 weeks post treatment with 3-dimensional conformal RT (3D-CRT) [8]. As such, the first assessment of treatment response is typically scheduled at this time-point through a combination of clinical and endoscopic examination with cross-sectional imaging. In practice, owing to their invasive nature, biopsies are usually reserved for cases where there are suspicious endoscopic or radiological findings. This imparts greater importance for imaging modalities such as MRI to pick up persistent or recurrent disease in a timely and accurate fashion.

Through this retrospective study, we explore the reporting patterns of post treatment MRI (ptMRI) for NPC patients, and aim to determine the optimal timing for the first ptMRI in a real-life clinical setting. In addition, we review current evidence and evaluate the ability of MRI compared to other clinical or radiological surveillance modalities in detecting locoregional recurrences (LRR).

## Methods

### Patients

Approval for this study was obtained from the Institutional Review Board (IRB). The NPC database, comprising all patients with histologically-proven NPC treated in two tertiary hospitals in Singapore, was retrospectively reviewed. Patients with non-metastatic NPC of any histological subtype treated with curative intent by RT alone, concurrent chemoradiation (CCRT) with or without induction chemotherapy between February 2013 and July 2017 were included for analysis. Pre-treatment evaluation for all patients included a complete history and physical examination, endoscopic assessment with biopsy, and staging scans. Computed Tomography (CT) or MRI was used for local staging, positron-emission tomography (PET)-CT was done to exclude metastatic disease. The staging system used was the American Joint Committee on Cancer (AJCC) 7th edition. Histology was classified according to the World Health Organisation system. For inclusion, at least one post-treatment MRI (ptMRI) needed to be done within 1 year of RT completion. Patients who did not complete the prescribed course of RT or have ptMRIs done and reported in other local/overseas institutions were excluded.

### Treatment

According to institutional guidelines, patients with stage I and node-negative stage II disease were treated with RT only. Node-positive stage II, stage III to IVB patients were treated with CCRT; those with T4 or N3 disease were also offered 2–3 cycles of induction chemotherapy. All patients received Intensity Modulated Radiotherapy (IMRT). CT simulation was done with administration of intravenous contrast; fusion with pre-treatment MRI was done for planning wherever possible. Treatment was carried out with patients in a supine position immobilised by a thermoplastic shell. Our treatment protocol closely follows that used in the Radiation Therapy Oncology Group (RTOG) 0615 trial [9]. All patients were prescribed a total dose of 69.96Gy in 33 fractions. Clinical Target Volume (CTV) is designated as the Gross Tumour Volume (GTV) with a circumferential margin of ≥5 mm; where tumour is in close proximity to critical structures such as the optic apparatus or brain stem this margin may be reduced accordingly. CTV_70_ included gross disease in the nasopharynx and any overtly involved lymph nodes. High risk and low risk subclinical regions as outlined in the RTOG 0615 radiotherapy schema were prescribed 59.4Gy and 54Gy respectively. Concurrent weekly cisplatin-based chemotherapy (30-40 mg/m^2^) was administered during RT. Treatment breaks were not specifically recorded.

### MR imaging, interpretation and timing

Timing of ptMRIs was calculated from the last day of RT. As per institutional practice, all scans were either reported or verified by a radiologist with a special interest in head and neck imaging. There were minor variations in the MRI protocol between the two tertiary hospitals but in all patients, four key sequences were performed: axial T1-weighted, axial T1-weighted contrast-enhanced fat-suppressed, axial T2-weighted and diffusion-weighted series.

The first ptMRI reports were reviewed and coded as ‘complete response’ (CR), ‘partial response’ (PR) or ‘indeterminate’ (ID). For the first ptMRI, CR is defined as complete resolution of disease or absence of any residual tumour in both primary and nodal sites in the radiology report. In subsequent ptMRIs, CR also included stable post-treatment changes seen on later imaging. PR is defined as the presence of residual disease in either the primary and/or affected nodes. Where the report indicated inability to definitively distinguish between residual tumour and post-treatment changes, or suggested clinical/endoscopic correlation, or repeat imaging, it was coded as ID.

To determine the optimal timing of first ptMRI, we arbitrated a cut-off point of 4 months +/− 15 days (106–135) compared with 3 months +/− 15 days (75–105). Based on current institutional practice, the first ptMRI is typically done at 3 months post RT. However, in our experience the rate of indeterminate outcomes is high when the scan is performed at this time point. We hypothesised that delaying the timing of the first ptMRI by 1 month (or 30 days), the proportion of ID ptMRIs may be reduced. This particular value was selected so as not to deviate too far from the common practice of 3-month post-RT scans, whilst keeping in mind that further delays to first ptMRIs may compromise timely detection of residual disease.

### Follow-up

Depending on extent of acute toxicity, patients were reviewed at weeks 2 and 4 post RT. Subsequently the follow-up schedule would be 2 to 3-monthly until end of year 2, 4-monthly in year 3 and 6-monthly in years 4 to 5. Post-treatment review comprises clinical and endoscopic examinations, and may be shared between the patient’s Ear, Nose & Throat (ENT) surgeon or medical oncologist where appropriate. Although there is a lack of specific guidelines with regards to the schedule of post treatment imaging, the first ptMRI was typically done at around the 3-month mark. Subsequent ptMRIs were not mandated if the first scan was reported as a CR; but were ordered where clinically indicated, for example in patients with PR/ID on first ptMRIs, or those with suspicious clinical or endoscopic findings. Patients with LRR based on ptMRIs were identified and reviewed to determine the timing of recurrence, how it was first detected (clinically versus radiologically), and how it was managed. The follow-up period was calculated from the last day of RT to day of last medical encounter or death.

### Statistical analysis

Descriptive analysis was done using frequency with percentage and median. Univariable Cox proportional hazard regression analysis was performed to look for association between various patient, disease and treatment characteristics with achieving CR on first and subsequent ptMRIs. Covariables with *P*-value of ≤0.05 in the univariable analysis were included in the multivariable Cox proportional hazard regression analysis to identify independent factors associated with CR. For all analyses, two-sided *P*-values of < 0.05 were considered to be statistically significant. Statistical analyses were performed using STATA version 14.0.

## Results

Two hundred sixty-two patients treated between February 2013 and July 2017 were eligible for analysis (Table 1). 196 (74.8%) were males and the median age at diagnosis is 55 (range 15–82). The most common histological subtype was undifferentiated non-keratinising (WHO Type III), in 243 patients (92.8%). 70 (26.8%) patients had stage I or II disease. Under TNM classification, 83 (31.7%) patients had T4 and 43 (16.4%) had N3 disease respectively. 51 (19.5%) patients received RT alone. The rest received chemotherapy in combination with RT: 124 (47.3%) underwent CCRT, 87 (33.2%) had induction chemotherapy followed by CCRT. All patients received IMRT to the full prescribed dose of 69.96Gy in 33 fractions.

**Table 1:** Patient and tumour characteristics

Patient characteristics		Tumour characteristics			
*Number of patients*	262	*Histology*	No. (%)	*T-stage*	No. (%)
*Age at diagnosis (range)*	55 (15-82) years	Non-keratinising undifferentiated	243 (92.7)	1	90 (34.4)
*Gender*	No. (%)	Non-keratinising differentiated	10 (3.8)	2	25 (9.5)
Male	196 (74.8)	Keratinising	2 (0.8)	3	64 (24.4)
Female	66 (25.2)	Others	7 (2.7)	4	83 (31.7)
*Ethnicity*	No. (%)	*AJCC Stage (7*^*th*^*edition)*	No. (%)	*N-Stage*	No. (%)
Chinese	207 (79.0)	I	23 (8.8)	0	38 (14.5)
Malay	31 (11.8)	II	47 (18.0)	1	90 (34.4)
Indian	4 (1.5)	III	80 (30.5)	2	93 (35.5)
Others	20 (7.7)	IV A/B	112 (42.7)	3	41 (15.6)

**Fig. 1:**
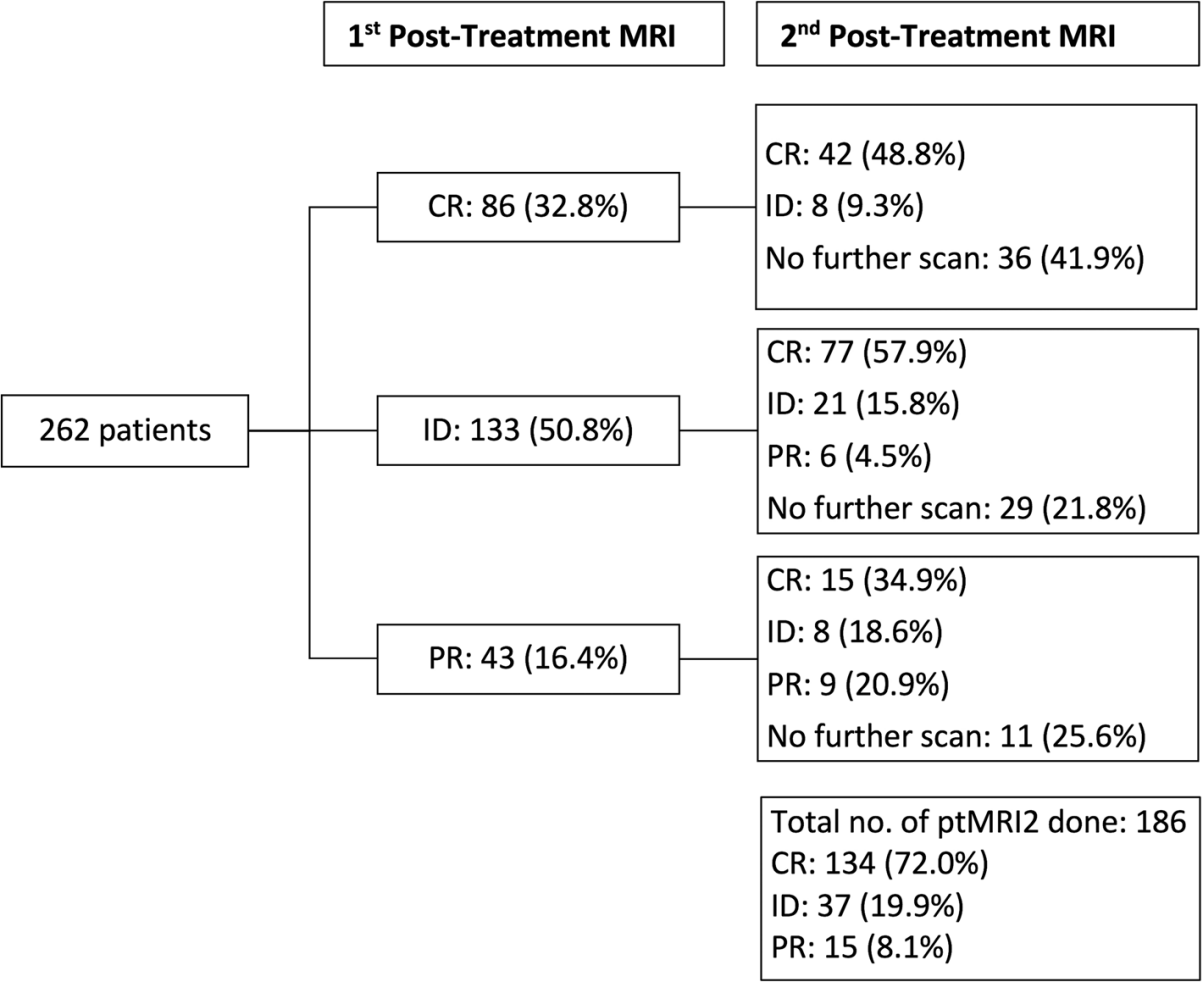
Post treatment MRI (ptMRI) responses. CR: Complete Response; ID: Indeterminate; PR: Partial Response

All 262 patients had at least one ptMRI. Of which, 86 (32.8%) were reported as CR, 133 (50.8%) as ID and 43 (16.4%) as PR. In the 133 patients whose first ptMRI was reported as indeterminate, 104 (78.2%) went on to have a second ptMRI (the remaining 29 patients had no further scans). In this group, 77 (57.9%) of the second scans were reported as CR, 21 (15.8%) remained ID and 6 (4.5%) were PR (Fig. 1).

Multivariable analysis showed that T1 stage and AJCC Stage I were significantly associated with achieving CR on the first ptMRI (Odds Ratio [OR] 2.96, *p* = 0.036 and OR 1.78, *p* = 0.046 respectively). Receiving CCRT was approaching significance (OR 2.46, *p* = 0.054) (Table 2).

**Table 2:** Factors associated with complete response (CR) on first post treatment MRI (ptMRI)

Variable	Univariate analysis			Multivariate analysis		
	OR	95% CI	P	OR	95% CI	P
Age	1.00	0.98–1.03	0.772			
Gender:						
Female	0.94	0.52–1.71	0.84			
Male (Ref)	1					
T stage:						
T1	3.63	1.82–7.20	< 0.001	2.96	1.07–8.13	0.036
T2	2.83	1.07–7.46	0.035	2.24	0.56–8.90	0.25
T3	1.79	0.84–3.85	0.134			
T4 (Ref)	1					
N stage:						
N0	1.87	0.76–4.56	0.136			
N1	0.90	0.42–1.94	0.86			
N2	0.62	0.28–1.36	0.24			
N3 (Ref)	1					
AJCC Stage:						
Stage I	4.2	1.67–10.51	0.002	1.78	0.39–8.11	0.046
Stage II	2.31	1.12–4.76	0.023	0.75	0.23–2.46	0.633
Stage III	1.29	0.68–2.44	0.433			
Stage IV (Ref)	1					
Treatment modality:						
RT alone	3.12	1.45–6.74	0.004	1.36	0.39–4.72	0.627
ChemoRT	2.51	1.32–4.78	0.005	2.46	0.98–6.16	0.054
Induction chemo (Ref)	1					

*OR* Odds Ratio, *CI* Confidence Interval, *AJCC* American Joint Committee on Cancer, *RT* Radiotherapy, *Ref* Reference

The median time from end of RT to having the first ptMRI was 93 days (range 32–346). ID status was more likely if the scan was done between 75 and 105 days (3 months +/− 15 days) compared to 106–135 days (4 months +/− 15 days) post RT (OR 2.13, 95% CI 1.16–4.12, *p* = 0.024) (Table 3).

**Table 3:** Factors associated with indeterminate (ID) status on first post treatment MRI (ptMRI)

Variable	Univariate analysis		
	OR	95% CI	P
Age	1.00	0.99–1.03	0.343
Gender:			
Female	1.57	0.89–2.76	0.119
Male (Ref)	1		
T stage:			
T1	0.98	0.54–1.77	0.934
T2	0.88	0.36–2.15	0.779
T3	1.02	0.53–1.95	0.964
T4 (Ref)	1		
N stage:			
N0	1.38	0.57–3.33	0.478
N1	1.71	0.82–3.59	0.154
N2	1.90	0.91–3.97	0.087
N3 (Ref)	1		
AJCC Stage:			
Stage I	0.74	0.30–1.81	0.509
Stage II	1.04	0.52–2.06	0.919
Stage III	1.33	00.75–20.37	0.329
Stage IV (Ref)	1		
Treatment modality:			
RT alone	0.90	0.45–1.79	0.759
ChemoRT			
Time to first ptMRI (days)			
75–105	2.13	1.16–4.12	0.024
106–135 (Ref)	1		

*OR* Odds Ratio, *CI* Confidence Interval, *AJCC* American Joint Committee on Cancer, *RT* Radiotherapy, *Ref* Reference

The median follow-up duration was 2.36 years (range 0.08–4.6). LRR was detected in 27 (10.1%) patients during follow-up. Of these, 20 (74.1%) were initially picked up by ptMRIs, 3 (11.1%) were detected on nasoendoscopy and 2 (7.4%) by PET-CT. In this group of patients, 16 (59.3%) had local recurrence, 7 (25.9%) had regional recurrence in neck nodes, 4 (14.8%) had synchronous local and regional recurrences.

## Discussion

Outcomes for patients with NPC have improved over the years with the introduction of chemotherapy and IMRT. However, local failure in the form of residual or recurrent disease still occur in 10–30% of cases [10, 11]. The assessment of treatment response and clinical surveillance after definitive therapy for NPC is important, in order to permit earlier recognition of local failure and initiation of salvage therapies. Typically, patients are assessed clinically with cranial nerve examination, neck palpation and endoscopic inspection, in combination with imaging such as CT, MRI or PET-CT. Any suspicious lesions are then biopsied to obtain histological confirmation.

Given that radiation can lead to anatomical distortion within the treatment field, identification of residual or recurrent disease is often challenging. Palpation for cervical lymph nodes may be limited by fibrosis of neck musculature. Post radiation endoscopic examination may only reveal subtle mucosal changes such as fullness of postnasal space (PNS), or a mass which may represent fibrosis, crust or slough rather than residual tumour [12], and submucosal or deep-seated recurrences may be missed. Sensitivity of endoscopic examination in detecting persistent disease after RT is only 40.4%. Similarly, endoscopic biopsies run the risk of sampling errors as residual tumour cells are often scattered in small clusters, resulting in a sensitivity at 6 weeks post RT of 59.3% [13].

Radiological assessment faces similar difficulties, and to date there is no consensus regarding the optimal imaging modality in the post treatment setting. The utility of MRI, whilst well established in the initial staging of biopsy-proven NPC, is less clear post treatment. Compared to CT, MRI is able to better differentiate post radiation changes from recurrent tumour and delineate extent of disease [14, 15]. The identification of skull base erosion is improved with contrast-enhanced fat-suppressed sequences [5].

However, when compared to PET-CT, MRI may be limited in its ability to distinguish between post RT changes often seen in the irradiated nasopharynx and neck e.g. tumour necrosis, tissue fibrosis and inflammation, from true viable tumour. Conversely, changes in tissue metabolism may precede changes in tumour morphology or volume. Liu et al in a systemic review concluded that PET-CT, with its ability for combined functional and anatomic assessment, had superior pooled sensitivity, specificity and overall diagnostic accuracy when compared to both CT and MRI [16]. PET-CT also has the added benefit of uncovering any systemic metastases within the same examination, which can impact on goals of further treatment.

Nevertheless, there are drawbacks to relying solely on PET-CT to uncover residual or recurrent NPC. Disease at the primary site is more accurately demonstrated on MRI rather than PET-CT (92.1% vs. 85.7%) according to Comoretto et al. [17]. The latter produced false negative findings especially where there is intracranial extension of disease. False positive results have also been associated with PET-CT when it is done too early post RT due to the residual inflammatory effects causing apparent increased glucose metabolism. It has been suggested that the PET-CT should be done 6 months or later post RT for optimal accuracy (sensitivity and specificity are 92 and 100% at 6 months or later vs 33 and 64% within 5 months) [18]. Additionally, cost and resource availability can be limiting factors for the prevailing use of PET-CT. In view of these considerations, it is likely that MRI and PET-CT should be complementary, in order to improve overall diagnostic accuracy for recurrent or residual disease [17]. Our data indicates that, within the boundaries of our institutional practice, most LRR are detected by MRI rather than non-MRI radiological modalities or clinical examination. If salvage surgery or RT is planned for LRR, the superior ability to determine extent of tissue invasion with MRI makes it preferable to PET-CT in guiding resectability or extent of re-treatment required.

Another issue to address is the optimal timing of MRI in view of the potential diagnostic uncertainties post RT. Guidelines suggest varying timing of post treatment imaging ranging from 3 to 12 months [19–21]. The time course of NPC regression after definitive treatment has been studied histologically and radiologically. In 1999 Kwong et al followed 803 NPC patients treated with 3D-CRT with or without induction chemotherapy, through 2-weekly endoscopic biopsies. 93.2% achieved histologic remission by 12 weeks post RT. [8] This formed the basis upon which many subsequent studies relied on when scheduling post treatment imaging. Li et al in 2017 challenged this paradigm with data from 556 NPC patients treated definitively with IMRT and followed up with serial MRIs [22]. All MRI scans were reviewed independently by two radiologists with extensive experience in head and neck cancer imaging. In this group, 83.3% of patients achieved a clinical complete response (cCR) – defined as no evidence of residual tumour or nodal disease based on examination with MRI and flexible nasoendoscopy - at 3–4 months (early cCR), a figure which increased to 91.4% at 6–9 months (delayed cCR). Interestingly, prognosis of patients with a delayed cCR was no different to those with an early cCR, leading the authors to conclude that 6–9 months may be the best time point for assessment of maximal tumour response to IMRT.

The increasing use of IMRT as standard of care, as well as CCRT has been associated with delayed primary tumour regression through mechanisms which to date remain unclear [23]. Whilst results from both Li’s and our study both suggest a lag time between histological and radiological tumour regression, a significantly lower proportion of patients in our series (32.8% vs 83.3%) achieved a radiological CR on the first ptMRI. This may be explained by the methodology of our study, which looks at the real-world radiology reports, rather than having one or more radiologists retrospectively reviewing imaging and coming to a binary outcome regarding the absence or presence of residual disease. We believe our method reflects real-life clinical practice more closely, where limits of certainty in imaging interpretation is accepted, and collective decision-making is undertaken in a multidisciplinary setting for indeterminate cases. In spite of this uncertainty, having the first ptMRI done at an earlier date may still be worthwhile in order to provide a crucial baseline which can inform future scans. In addition, there may be a window period between 3 and 4 months and 6–9 months where further investigations can lead to earlier diagnosis of residual or recurrent disease, permitting prompt initiation of salvage therapies. Indeed, our study offers evidence that the optimal timing of the first ptMRI should be 4, rather than 3 months to reduce the rates of uncertainty in radiology reports.

We acknowledge that this study has some limitations. Firstly, the retrospective design meant that patients were not followed up based on a standardised protocol. ptMRIs were done at the clinicians’ discretion, resulting in a wide date range for the first (32–346 days) and subsequent ptMRIs. Of note, a proportion of patients who had ID/PR status did not go on to have subsequent ptMRIs, reflecting the pattern of real-life clinical practice. Depending on overall clinical suspicion of residual or recurrent disease, these patients may have undergone investigation with other imaging modalities such as PET-CT or had biopsies which confirmed or excluded presence of disease, thereby sidestepping the need for further serial MRIs. Secondly, the determination of ptMRI status was based primarily on the authors’ review of actual radiology reports rather than centralised review by dedicated radiologist(s). Although the ptMRIs are all reported by radiologists with a special interest in head and neck imaging, their level of experience may be differing.

## Conclusion

MRI has an important role in NPC surveillance compared to other imaging modalities and detected a majority of loco-regional recurrence in our series. However, most patients will require more than one ptMRI to reach a definitive status. The rate of ID scans may be reduced by delaying the first ptMRI to 4 months post-RT.

## Data Availability

The datasets generated and/or analysed during the current study are not publicly available as presently we have not been granted permission by the institutional review board to do so. However, data can be made available from the corresponding author on reasonable request.

## Abbreviations

3D-CRT: 3-dimensional conformal radiotherapy
AJCC: American Joint Committee on Cancer
CCRT: Concurrent chemoradiation
CR: Complete response
CT: Computed tomography
ID: Indeterminate
IMRT: Intensity-modulated radiotherapy
LRR: Locoregional recurrence
MRI: Magnetic resonance imaging
NPC: Nasopharyngeal carcinoma
PET: Positron emission tomography
PR: Partial response
ptMRI: Post treatment magnetic resonance imaging
RT: Radiotherapy
